# Restricted working hours in Austrian residency programs: Survey results

**DOI:** 10.1007/s00508-018-1340-1

**Published:** 2018-04-27

**Authors:** Konstantin D. Bergmeister, Martin Aman, Bruno K. Podesser

**Affiliations:** 1 CD Laboratory for the Restoration of Extremity Function, Department of Surgery, Medical University of Vienna, Vienna, Austria; 2 Ludwig Boltzmann Cluster for Cardiovascular Research at the Center of Biomedical Research, Medical University of Vienna, Spitalgasse 23, 1090 Vienna, Austria

**Keywords:** Residency programs, Working hour restrictions, Survey on new working hours regulations, Austrian survey, Arbeitszeitgesetz Österreich

## Abstract

**Background:**

New regulations for working hours of medical doctors have been implemented in Austria based on the European directive 2003/88/EG, limiting on-duty working hours to 48 h per week. Clinical work is, therefore, substantially reduced compared to previous decades, and little is known on physician and students’ opinions on this matter. We illustrate survey results concerning on-job training, its difficulties, and implications for restricted working hours.

**Methods:**

We conducted an internal survey among M.D. and Ph.D. students and medical staff members at the Medical University of Vienna using the MedCampus system (CAMPUSOnline, Graz, Austria) and SPSS (V.21, IBM Corp, Armonk, NY, USA).

**Results:**

Participants were 36.5% staff members and 63.5% students. Students rated continuous education of physicians high at 9.19 ± 1.76 and staff members at 8.90 ± 2.48 on a 1–10 (1 unimportant, 10 most important) scale. Students rated limited time resources, while staff considered financial resources as the greatest challenge for in-hospital education. Overall, 28.85% thought that restricted working hours can positively influence education, while 19.04% thought the opposite and 52.11% were undecided.

**Discussion:**

Considering the limited available time and financial resources, education of tomorrow’s medical doctors remains an important but difficult task. While participants of our survey rated education as very important despite its many challenges, the opinions towards limited working hours were not as clear. Given that over 50% are still undecided whether reduced work hours may also positively influence medical education, it clearly presents an opportunity to include the next generations of physicians in this undertaking.

## Introduction

New regulations for working hours of medical doctors have been implemented in all 27 European member countries of the EU based on the European directive 2003/88/EG, effectively limiting physicians on-duty to 48 h per week. This regulation was accordingly implemented over the past years into Austrian law and has ever since reshaped the concept of on-duty hours in Austrian hospitals, where traditionally 90 h were considered the norm [1].

Since the introduction of reduced working hours, there has been a vital debate about the benefits and disadvantages of this measure. Some consider this reduction in working hours a positive trend towards patient safety [2, 3] and employee-friendly working hours in line with modern concepts of work-life balance [4]. Based on several studies, reduced working hours have been shown to prevent secondary mental diseases [5] and reduce the overall high suicide rate of medical doctors [6, 7]; however, others criticize the negative effects on residency training, as some analyses on surgical training indicate that restricted operative exposure can negatively impact surgical performance and thus patient outcomes [8, 9]. These effects are thought to be further aggravated by the steep learning curves of many novel surgical techniques, limited teaching resources in times of high cost pressure, more complex cases and generally sicker patients [10].

Despite this vital ongoing debate about the pros and cons of reduced working hours on patient safety and teaching matters [11], there is little knowledge of doctors and students’ opinion on this matter. We believe that this may, however, be a vital component in this discussion to positively shape the future of this profession in terms of well-educated doctors, patient safety and work-life balance. In this study we present first survey results of Austrian hospital staff members and students concerning on-job training, its difficulties, and implications on restricted working hours.

## Methods

We designed an in-house survey with several multiple choice questions to investigate the opinions on medical training and the current situation with respect to the new working hours. This survey was conducted using the MedCampus system (CAMPUSOnline, Graz, Austria) of the Medical University of Vienna, and included a total of 10,335 M.D. and Ph.D. students and 3824 staff members. The survey preparations were conducted from January to October 2015, with the survey being accessible from October to December 2015. The analysis took place from November 2015 to June 2017. Statistical analyses were conducted using Microsoft Excel and SPSS (V.21, IBM Corp, Armonk, NY, USA). Prior to the survey, approval was obtained from the data privacy committee of the Medical University of Vienna.

## Results

In our in-house survey, a total of 906 participants completed all relevant questions. This accounts for an 6.38% overall response rate of all students and staff members. The study’s participants were 36.5% staff members and 63.5% MD and PhD students. Overall, students rated continuous education of medical doctors at 9.19 ± 1.76 and staff members at 8.90 ± 2.48 on a 1–10 (1 unimportant, 10 most important) scale, indicating its importance to the participants.

**Fig. 1:**
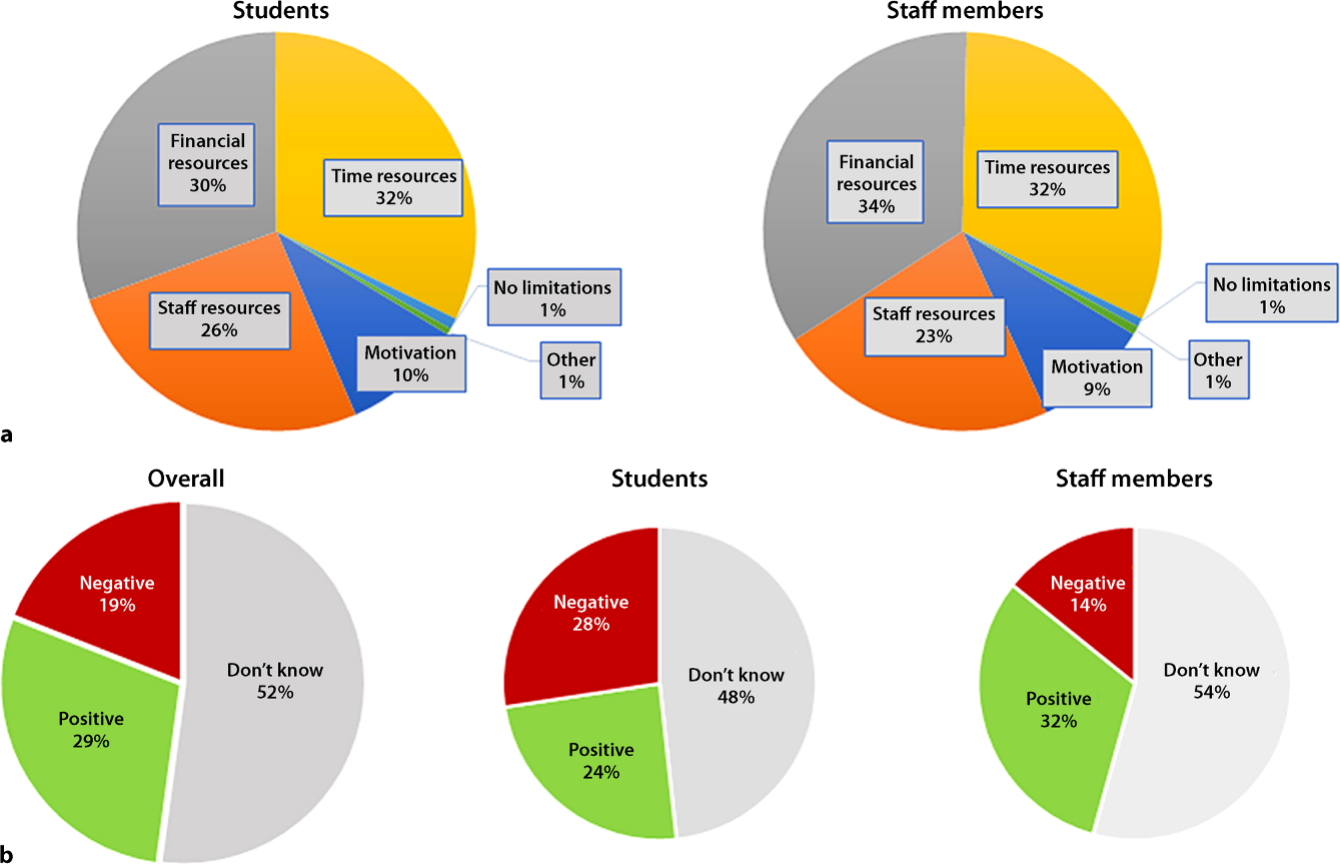
**a** Challenges for in-hospital education as rated by students and staff members. Students considered time resources as the biggest challenge, whereas staff members considered financial resources as the biggest challenge. **b** “Can restricted working hours positively influence medical education?”: Overall 29% thought this is possible, whereas 19% did not. This number was higher in staff members (32%) than in students (24%). A high number of participants did not know whether a positive or negative effect would result

Students (32%) considered limited available time, while staff (34%) considered limited financial resources as the greatest challenge for in-hospital education. Limited personnel, financial and time resources accounted for 80% in both groups. Less than 1% of either students or staff members believed there are no limitations for on-job training in Austrian hospitals (Fig. 1a).

Overall, 28.85% thought that restricted working hours can positively influence medical education, while 19.04% thought the opposite and 52.11% were undecided. The percentage of participants that were not sure if the new working hours could positively influence education, was higher in staff members (54%) compared to students (48%). Likewise, more staff members believed in a positive change (31%) compared to students (24%) (Fig. 1b).

## Discussion

Reduced working hours and the effects on the medical profession continue to be a highly discussed matter [3, 12–15]. Despite the vital debate between advocates and opponents of this change, our survey indicated that the majority of affected students and staff members are still uncertain if this trend is positive or negative for medical education. Therefore, a large opportunity is ahead to include affected doctors as well as the next generation of doctors and mutually shape our professional environment accordingly. Clearly, a major task in this undertaking is to ensure that the reduction in clinical exposure does not negative influence the quality of our work. A number of publications have indicated that the current duration of residency programs may not be able to provide the same level of education with reduced working hours [8, 9]. Although these results are not to be considered as final [14, 15] they should, however, alert us to find possibilities to compensate for any possible loss in quality. This may involve the further outsourcing of administrative tasks in order to focus the work of medical doctors on treating patients. Other approaches test the use of artificial intelligence to help doctors in the diagnosis and treatment of diseases or simply reduce tasks that do not require human interaction [16, 17]. Furthermore, teaching has and will further evolve to transfer medical knowledge as well as procedures or surgeries in a faster pace to medical trainees. This is achieved using modern media, training workshops and realistic computer or in vivo simulations [18–24]. Also, medical schools have adapted to the need of training medical doctors ready for clinical work, by shifting from a theoretical learning approach to bedside teaching and early involvement in the clinical routine (Practical clinical year, “Klinisch Praktisches Jahr”; [25, 26]). Some even consider extending residency programs with a current duration of (mostly) 6 years to longer periods to compensate for the loss of clinical exposure.

In this study, one limiting factor is the general response rate in our survey, which was, however, comparable to other medcampus surveys at our institution. As indicated by the high number of undecided students and staff members, we believe it is necessary to focus on the future of medical education now. It is unlikely that regulations regarding working hours will change to traditional levels and thus medical doctors have to identify solutions for this challenge early before a dramatic loss in quality occurs. In our study, financial, staff as well as time resources were considered to be equally challenging for medical education. All need to be addressed to provide better education either by an increase in budget for external resident training or for staff to compensate for training absences. Obviously, these matters involve high costs for hospital providers and demand a political motivation to invest in our healthcare system. Some federal states in Austria have already been forced to provide more staff and resources for training to compete with surrounding countries for qualified staff members. The political weight of such developments and the possible decline of our healthcare system is significant. Therefore, this agenda has emerged in recent electoral campaigns, both national and international, with increasing frequency and attention.

If we gather this momentum to shape medical education and the environment we work in, we may very well be able to compensate for less time on duty without compromising the quality of our work. Furthermore, we can continue to ensure the trust of our patients not only in our work but in the future of our medical system. This may however require a united approach to inform the public and politicians of the challenges ahead and their implications on our healthcare system.

## Conclusion

Considering the limited available time and financial resources, education of tomorrow’s medical doctors remains an important but difficult task. While participants of our survey likewise rated education as very important despite its many challenges, the opinions towards limited working hours were not as clear. Given that over 50% were still undecided whether reduced working hours can also positively influence medical education, it clearly presents an opportunity to include the next generations of physicians in this undertaking.
