# Correction to: Restricted working hours in Austrian residency programs: Survey results

**DOI:** 10.1007/s00508-018-1349-5

**Published:** 2018-05-23

**Authors:** Konstantin D. Bergmeister, Martin Aman, Bruno K. Podesser

**Affiliations:** 1 CD Laboratory for the Restoration of Extremity Function, Department of Surgery, Medical University of Vienna, Vienna, Austria; 2 Ludwig Boltzmann Cluster for Cardiovascular Research at the Center of Biomedical Research, Medical University of Vienna, Spitalgasse 23, 1090 Vienna, Austria

**Correction to:**

**Wien Klin Wochenschr 2018**

10.1007/s00508-018-1340-1

The original version of this article unfortunately contained a mistake. The presentation of the sentence “limiting on-duty working hours to 58h per week.” was incorrect. The correct limitation of the on-duty working hours is 48 h per week. The original article has been corrected.

